# A Case of Malignant Pleural Mesothelioma With Unknown Asbestos Exposure

**DOI:** 10.7759/cureus.69966

**Published:** 2024-09-23

**Authors:** Ani Paremuzyan, Ewele Onwubuya, John Mathews

**Affiliations:** 1 Internal Medicine, California Hospital Medical Center, Los Angeles, USA; 2 Oncology, Medical City Dallas Hospital, Dallas, USA

**Keywords:** asbestos, effusion, exudate, lung, malignant, mesothelioma, plaques, pleural, screening, thoracotomy

## Abstract

Malignant pleural mesothelioma (MPM) is a rare, locally invasive tumor that develops from mesothelial cells lining the lung's pleura. It is mostly associated with prolonged asbestos exposure. The long latency period between asbestos exposure and clinical symptoms makes diagnosing MPM challenging. This report describes a 57-year-old Hispanic female who presented with a persistent nonproductive cough and was ultimately diagnosed with advanced-stage pleural mesothelioma after extensive work-up. It highlights the difficulties in diagnosing MPM in patients without apparent asbestos exposure independent of age or gender.

## Introduction

Malignant pleural mesothelioma (MPM) is a rare yet aggressive cancer of mesothelial cells of the parietal pleura lining the lungs, 70-90 % of which show a link with occupational asbestos exposure [1,2]. Asbestos inhalation causes chronic inflammation that activates proto-oncogenes and generates free radicals. Notably, although the link between asbestos exposure and the development of MPM has been well established, there have been cases where females and a certain number of males develop MPM without a definitive history of such exposure. These cases can be attributed to various genetic factors (e.g., germline mutations of BRCA 1 Associated Protein (BAP1)), environmental exposure to naturally occurring minerals such as erionite, non-occupational exposure, or other risk factors (e.g., ionizing radiation therapy) that are currently not well understood [2].

The initial clinical manifestations of the disease typically occur 30-40 years after the first exposure. According to the CDC, it primarily targets males after their fifth decade of life, with 72 being the average age at diagnosis. The prevalence of MPM is higher in white and Hispanic populations when compared to Asian or Black populations. Typical symptoms at first presentation include dyspnea and non-pleuritic chest pain. Only 10% of patients present atypically with weight loss, fatigue, loss of appetite, cough, hemoptysis, and mediastinal mass [3,4]. The disease mortality rate is high, and median survival after diagnosis is under a year [5]. Interestingly, the annual number of deaths of females in healthcare and homemaker occupations has increased from 489 in 1999 to 614 in 2020 [6].

Due to its availability, plain chest radiography is the first imaging modality, with a finding of unilateral pleural effusion in 30-80% of patients, in addition to pleural plaques seen in only 20% of cases. Contrast-enhanced computed tomography (CT) is the next modality, with the most frequent finding of pleural thickening in more than 90% of patients [7]. Positron emission tomography (PET) is used to detect metastatic disease but has suboptimal sensitivity and specificity for staging MPM [8]. To confirm the diagnosis, a radiological guided or surgical biopsy is needed. MPM is non-curable. Standard first-line chemotherapy that improves mortality in MPM patients combines pemetrexed and cisplatin/carboplatin with Vitamin B12 and folic acid supplementation. It has an average response rate of 24% [9]. Surgery is a choice in the initial stages of the disease, yet it is associated with high morbidity and mortality due to complications [9].

## Case presentation

**Figure 1:**
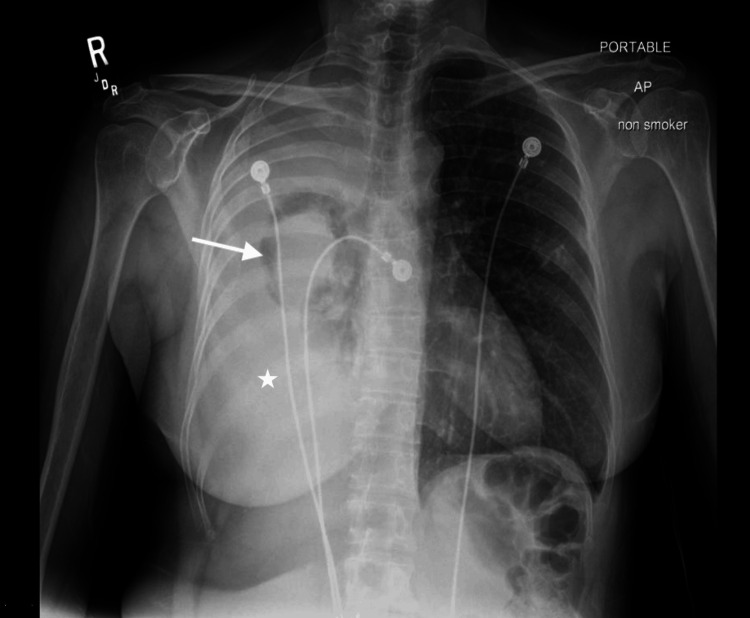
Single front-view chest X-ray: pleural effusion (white asterisk) and cavitary lesion (white arrow)

**Figure 2:**
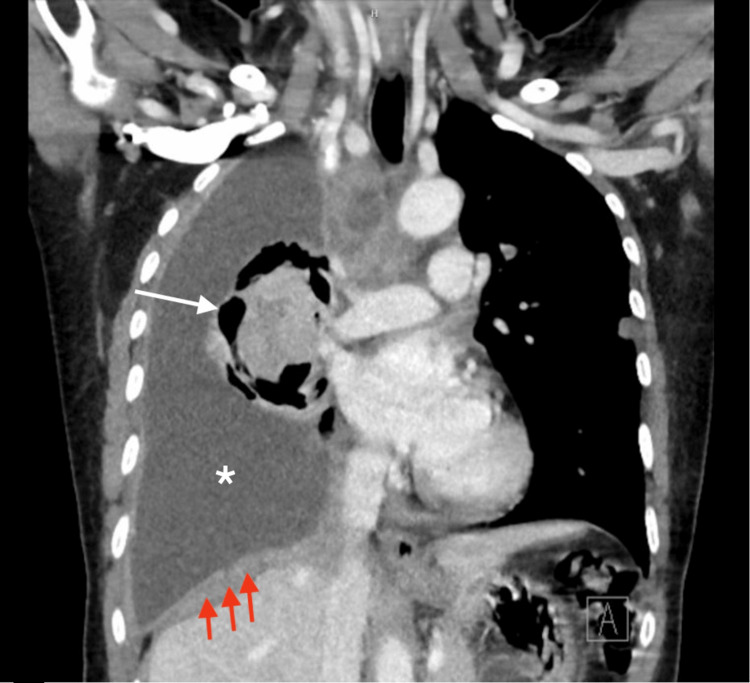
CT chest with contrast coronal view: irregular pleural thickening (red arrows), pleural effusion (white asterisk), and cavitary lesion (white arrow)

**Figure 3:**
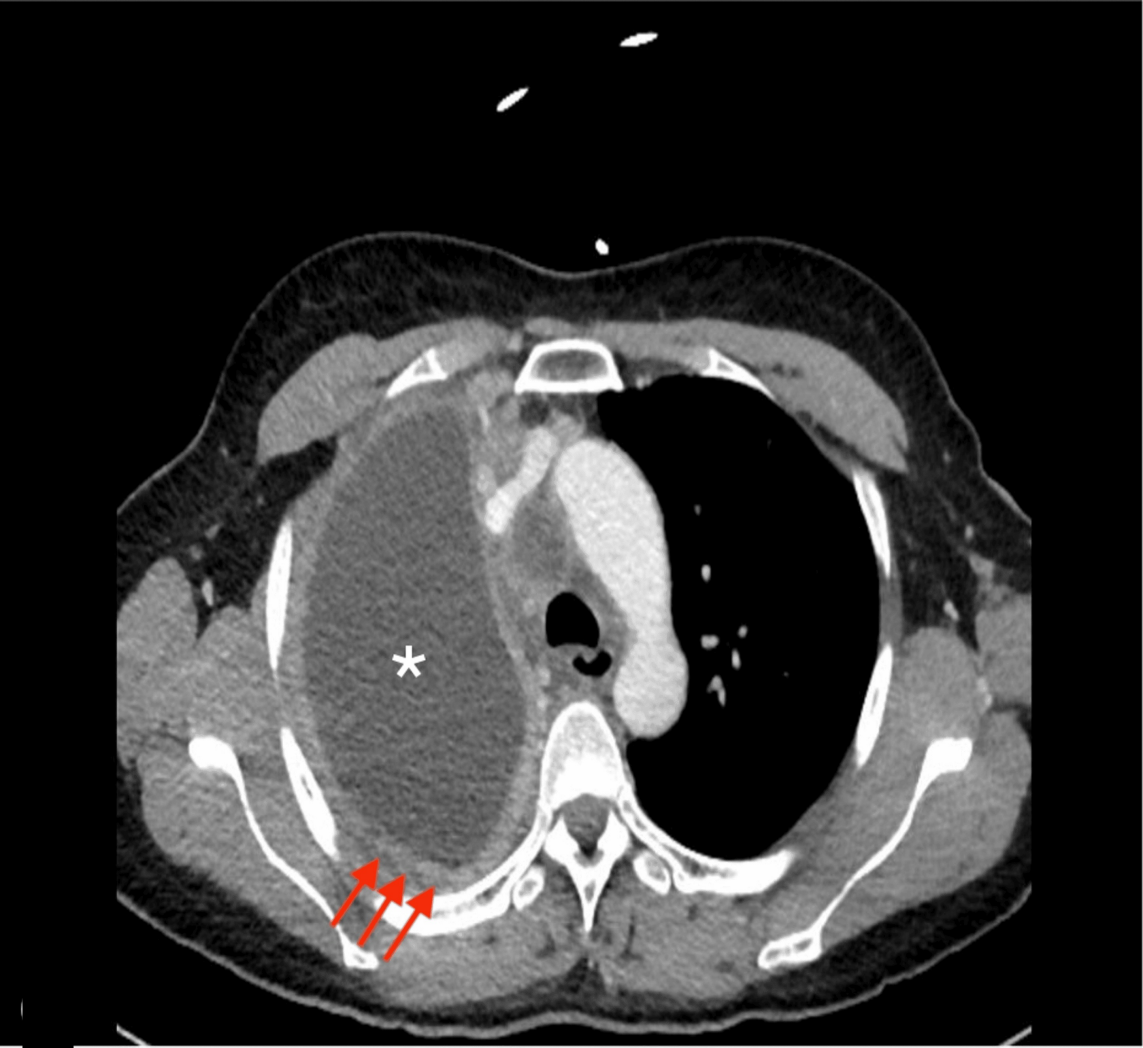
CT Chest with contrast axial view: irregular pleural thickening (red arrows) and pleural effusion (white asterisk)

A 57-year-old Guatemalan female with no significant PMHx presented to the emergency department for evaluation of a persistent cough and globus sensation for three months. She was initially seen by her primary care provider and was prescribed Symbicort with no improvement in her symptoms. She complained of fatigue, dyspnea on exertion, and unintentional weight loss over two weeks.​ She denied fevers, chills, nausea, hemoptysis, night sweats, joint/bone pain, and other constitutional symptoms. ​Family history was significant for lung cancer in her father at the age of 60. The patient denied any history of tobacco use. She was recently employed as a childcare worker; however, she had a significant history of seven years of employment as a housecleaner and a factory worker constructing metal cabinets. The patient was afebrile, tachycardic, and hypertensive, with a SpO2 of 98% on room air. Her physical examination was significant for right-sided inspiratory crackles and decreased breath sounds throughout the right lung. Procalcitonin and platelets were 0.22 and 456, respectively; all other labs were within normal limits. Chest X-ray (CXR) showed right-sided pleural effusion, near complete collapse of the ipsilateral lung, and cavitary lesion in the right mid-lung (7.1 cm) (Figure 1). Contrast CT was significant for right-sided pleural effusion, irregular pleural thickening (>7mm), large centrally necrotized right paratracheal node (2.1 cm), and calcified pleural plaques of the left lung (Figures 2, 3).​

Differential diagnoses included tuberculosis (TB), empyema, coccidiomycosis, pneumocystis pneumonia (PCP), and mesothelioma.​​Various procedures were performed, including blood cultures, AFB, and staining. The patient was placed in TB isolation and received empirical RIPE therapy (rifampin (RIF), isoniazid (INH), pyrazinamide (PZA), and ethambutol (EMB)) until TB was ruled out. The patient underwent video-assisted thoracotomy surgery with pleural exudate evacuation, pleural resection, decortication, and multilevel intercostal nerve block. The pathology report from the right pleural frozen sections and pleural exudate showed findings consistent with advanced-stage mesothelioma positive for calretinin, podoplanin (D2-40), and CK5/6 (Table 1). Podoplanin is a mucin-like glycoprotein that pathologists commonly use to differentiate mesothelioma from bronchogenic cancer in females with no smoking history [10]. Clinically distinguishing between the two types of cancer is crucial for accurate diagnosis, treatment planning, and prognosis assessment.

**Table 1:** Immunohistochemical data

Stain	Results
Calretinin	Highlights tumor cells
CK 5/6	Highlights tumor cells
D2-40	Highlights tumor cells and vessels
CK7	Highlights tumor cells
WT1	Highlights tumor cells and vessels
CK7	Negative
CK19	Highlights tumor cells
CD3	Highlights reactive T cells
CD20	Highlights reactive T cells
P40	Negative
P63	Negative
TTF-1	Negative
CK20	Negative
CD34	Highlights vessels and nonspecific stromal staining
CD68	Highlights histiocytes
CDX2	Negative
CEA(M)	Negative

The patient was discharged home with outpatient chemotherapy arrangements but, unfortunately, passed away a month later due to disease progression and associated complications. 

## Discussion

The incidence of MPM is rare, and diagnosis in its early stages is even more uncommon. In one case report, the patient had an incidental CT finding of extra pleural mass with smooth borders and thickened parietal pleura. CT-guided biopsy confirmed MPM. Treatment included surgical tumor resection that improved patient mortality significantly. The patient passed away 29 months (about two and a half years) later from tumor recurrence [11]. On the other hand, the patient in our case report presented with diffuse mesothelioma, a far more common observation than localized early-stage mesothelioma, when palliative measures are the only option and life expectancy is only 4-6 months. Diagnosis at an advanced stage is mainly due to diagnostic difficulties found in mesothelioma, such as the inability to detect the presence of neoplastic cells in cytological examinations of pleural effusions. 

Asbestos is a highly persistent hydrated magnesium silicate fiber. The leading theory is that asbestos fibers interfere with mitotic spindles during the cell cycle, leading to chromosomal abnormalities. Research demonstrates that sporadic mutations of chromosomes 1,3 and 22 were highly associated with malignant mesothelioma [12,13]. In addition, studies indicate asbestos exposure in sheet-metal workers who handled asbestos-containing materials without proper protection [14]. As opposed to other case presentations where mesothelioma cases were associated with asbestos exposure in males at the presenting age of 60-80 years old, our patient was a female in her fifties without known asbestos exposure. There has been minimal research explaining the association between middle-aged females with no known exposure to asbestos and the development of MPM. The absolute deviation from the accepted normal emphasizes that there is insufficient information on the risk factors of the disease due to misdiagnosis or loss of cases due to high mortality. More studies on the topic will help to differentiate the patients with the MPM better and sooner.

Differentiating between other pulmonary pathologies, including TB, empyema, coccidioidomycosis, and PCP, is essential when evaluating patients with significant respiratory symptoms. Empyema is confirmed through imaging and pleural fluid analysis via thoracentesis. TB is diagnosed with skin tests, CXR, and sputum cultures. The diagnosis of PCP is made through clinical suspicion and laboratory analysis of respiratory specimens, typically in immunocompromised individuals. Coccidioidomycosis is diagnosed through serology and cultures, especially in endemic regions.

Diagnosing MPM at an early stage is the key. Patients diagnosed sooner in the disease course are usually good candidates for surgery and, thus, have a five-year relative survival rate closer to 25%. On the other hand, patients diagnosed at an advanced stage of the disease have a five-year relative survival rate of less than 16% [15]. Moreover, a systematic approach combining practical screening methods in high-risk populations, detailed patient history, and targeted respiratory testing is essential for more accurate diagnosis and effective treatment options [16]. 

## Conclusions

Malignant mesothelioma is a rare yet aggressive cancer. The number of incidents is currently low due to theories of its association with past asbestos exposure. However, the prevalence of MPM may increase when considering other associations, such as chromosomal abnormalities. This case underscores the need for constant vigilance and maintaining a high suspicion for MPM in patients presenting with nonspecific pulmonary symptoms with any history of working in high-risk occupations.
